# Falls on an inpatient rehabilitation spinal injuries unit: the characteristics, circumstances, and consequences

**DOI:** 10.1038/s41393-022-00861-3

**Published:** 2022-10-22

**Authors:** Kathryn Marshall, Jennifer Fleming, Sridhar Atresh, Justin, R. Scott, Louise Gustafsson, Freyr Patterson

**Affiliations:** 1 School of Health and Rehabilitation Sciences, The University of Queensland, Brisbane, QLD Australia; 2 Department of Occupational Therapy, Princess Alexandra Hospital, Brisbane, QLD Australia; 3 Queensland Spinal Cord Injuries Service, Division of Rehabilitation, Princess Alexandra Hospital, Brisbane, QLD Australia; 4 School of Medicine, The University of Queensland, Brisbane, QLD Australia; 5 QCIF Bioinformatics, Institute for Molecular Bioscience, The University of Queensland, Brisbane, QLD Australia; 6 School of Health Sciences and Social Work, Griffith University, Brisbane, QLD Australia; 7 The Hopkins Centre, Menzies Health Institute Queensland, Griffith University, Brisbane, QLD Australia

**Keywords:** Rehabilitation, Patient education

## Abstract

**Study design:**

Retrospective audit

**Objectives:**

To describe the nature of falls and fallers in a spinal injuries unit (SIU) and identify factors associated with having more than one fall (recurrent fallers) and falls with physical or psychological consequences (consequential falls).

**Setting:**

An Australian inpatient rehabilitation SIU.

**Methods:**

Data were retrospectively extracted from falls incident reports and electronic medical records over a 5-year period. Data were analysed descriptively to summarise participant and fall details. Univariate analyses identified candidate variables for further investigation in a multivariate model for recurrent fallers and consequential falls.

**Results:**

Of the 566 persons admitted to the SIU, 132 (23%) participants experienced 207 falls over the 5 years. Of the fallers, 41 (31%) were recurrent fallers experiencing between 2 and 7 falls and 78 (59%) experienced a consequential fall. No significant variables were identified for recurrent fallers. For consequential falls, older age (OR = 1.038, 95% CI, 1.012 to 1.064, *p* = 0.004*)* and female gender (OR = 3.581, 95% CI, 1.269 to 10.103, *p* = 0.016*)* were significant, as well as falls that occurred on a Sunday (OR = 0.196, 95% CI, 0.061 to 0.630, *p* = 0.006). Falls while transferring were less likely to be consequential (OR = 4.100, 95% CI, 1.706 to 9.856, *p* = 0.002).

**Conclusions:**

Nearly one quarter of SIU inpatients experienced a fall with almost a third of those who fell experiencing recurrent falls. Older age, female gender, and Sundays were risk factors for falls with consequence.

## Introduction

Falls during a hospital stay increase the length and cost of the admission [1] and have physical and psychological consequences for individuals [2]. People with a spinal cord injury (SCI) often experience motor and sensory changes increasing risk of falls [3], particularly while learning their limitations and developing new skills during inpatient rehabilitation. Reported fall rates for persons with a SCI in neurorehabilitation wards are as high as 24% [4]. These rates suggest it is vital that fall prevention begins during inpatient rehabilitation [5] and highlights the need for understanding the nature of falls during this phase of recovery.

Research on the circumstances and consequences of falls for the SCI population has increased over the past 10 years with a large amount of the literature focused on persons living in the community [6, 7]. In the community, wheelchair users fall most commonly while transferring or wheeling in their wheelchair over uneven ground [8–10], and ambulant persons with SCI fall most commonly while bending [9, 11] or walking [11, 12]. It is common for people with SCI in the community to experience more than one fall (termed recurrent falls) [8, 10, 11, 13] and to experience consequences or harm such as pain, bruises, cuts, fractures and loss of consciousness from falling (termed consequential falls) [8–12]. While research on falls in the community is valuable, the circumstances of falls, and the prevalence of recurrent and consequential falls in inpatient SCI rehabilitation is largely unknown. Furthermore, the characteristics of recurrent fallers and types of falls that are consequential are yet to be explored during inpatient admission.

Wilson et al. [4] compared inpatient falls for SCI, acquired brain injury and neuromusculoskeletal populations although additional data for falls of patients with SCI is needed. Understanding the specific circumstances of falls during SCI rehabilitation, may help develop targeted and individualised fall prevention programs early in the rehabilitation journey [2, 14, 15]. Therefore, this study aimed to describe the nature of falls and fallers in inpatient SCI rehabilitation, and identify factors associated with recurrent falls and consequential falls. Specific objectives were to 1) describe characteristics of people who fall during inpatient SCI rehabilitation, 2) identify factors associated with recurrent fallers versus single fallers, 3) identify factors associated with people who experience consequential falls versus those who do not, 4) describe the circumstances and consequences of all falls reported during inpatient rehabilitation and 5) identify significant differences in characteristics of consequential falls versus non- consequential falls.

## Methods

This was a retrospective quantitative audit of records over a 5-year period on the spinal injuries unit (SIU) at the Princess Alexandra Hospital, a major tertiary hospital in Queensland, Australia. The SIU is a 40 bed, inpatient, specialised service which forms part of the Queensland Spinal Cord Injuries Service (QSCIS) and offers rehabilitation services to people with both traumatic and non-traumatic injuries.

Ethical approval was obtained from relevant hospital and university ethics committees. Eligible participants were identified through an audit of the incident reporting system (Riskman or PRIME) for the period Jan 2016 to Dec 2020. Participants were eligible if they were admitted to the SIU between 2016 and 2020 and experienced one or more falls documented in a fall incident report. At our hospital for the purpose of incident reporting, a *fall* was defined as “an unexpected event in which the participants come to rest on the ground, floor, or lower level.”[16] p1619]

### Data collection

Data were extracted from incident reports and medical records. Variables for data extraction were identified from existing literature reviews of falls in people with SCI [6, 7] and recommendations on utilisation of incident reports to analyse falls data [17]. Data extracted from the incident report included age and gender, date of fall, day of the week, time of fall and description of the event. Further data extracted from the medical record included date of admission and discharge, the cause of SCI, level of SCI, the participant’s American Spinal Injury Association (ASIA) Impairment Scale score [18] at rehabilitation admission and discharge, the participant’s primary means of mobility at the time of the fall, whether the incident was witnessed by staff, and the description of the event by health professionals involved. Participants cleared to walk on the ward at the time of the fall by the most recent physiotherapy chart entry were classified as ambulant. If a participant experienced recurrent falls, the most frequently reported mobility status was used. Consequences of the fall were retrieved from the medical record in the week following the fall. A serious consequence was classified as a fall that resulted in a fracture, head injury or internal injury [19] with all other consequences defined as minor. Participants were classified as having a consequential fall if any of their falls had a physical or psychological consequence documented. The categories of consequences were decided in consultation with the Director of the SIU (SA).

The audit was conducted by the first author (KM), a clinician with 5-years experience in SCI rehabilitation and familiar with the hospital’s medical record. A spreadsheet was created in Excel (Version 2111) and a detailed coding manual was developed with instructions for recording each variable to ensure consistency [20]. Accuracy of coding data into the spreadsheet was confirmed with the nursing educator working on the SIU for the first ten percent of the reports.

### Data analysis

The data were imported into the IBM Statistical Package for the Social Sciences (SPSS, Version 27) for data analysis.

Data were analysed descriptively using median and interquartile range (IQR) as continuous data were not normative. Frequencies and percentages were used for categorical data.

A series of tests were completed to identify significant person (age, gender, cause of SCI, level of SCI, ASIA impairment scale, ambulatory state) and fall-related (day of the week, month, year, season, time of day, time in admission when fall occurred, witnessed by staff, activity and location) variables to be included in the multivariable model to predict recurrent and consequential fallers and consequential falls. They included binary logistic regression for continuous variables (age) and Pearson’s Chi-squared tests – asymptotic significance (2 sided) or Fisher exact tests when expected cell count was less than 5, for remaining categorical variables. The level of significance was set at 0.05 except when significance was found for a non-binary variable (day of the week, activity, and location), and in this instance post hoc analysis was completed with level of significance adjusted to 0.01 using the Bonferroni method, rounded up to the nearest decimal place. Significant participant variables (age, gender) and fall variables (day of the week, witnessed by staff, transfers and bedspace) were entered into a multivariate generalised linear model with a binomial distribution weight for inverse number of falls for participant analysis and inverse maximum number of falls for fall analysis. Variables were retained in the model if the significance level was less than 0.05. In the falls analysis, whether the fall was experienced by a single or recurrent faller was included as a surrogate to account for correlations between falls within participants.

## Results

### Characteristics of fallers

There were 566 patients admitted to the SIU of which 135 (24%) were females. Over the 5-year period, 132 people (23% of the total number of admissions) experienced at least one fall incident. Table 1 reports the characteristics of fallers.

**Table 1:** Characteristics of fallers in inpatient SCI rehabilitation (*N* = 132).

		Falls			Consequence		
*n* (%)	All fallers	Single fallers	Recurrent fallers	*p*	Non-consequential	Consequential	*p*
		91 (69)	41 (31)		54 (41)	78 (59)	
Age, Median (IQR)	51 (27)	53 (28)	47 (26)	0.370^b^	46.5 (27)	55.5 (28)	0.025^b^
Gender, *n* (%)							
Male	102 (77)	67 (51)	35 (27)	0.136	47 (36)	55 (42)	0.026
Female	30 (23)	24 (18)	6 (5)		7 (5)	23 (18)	
Cause, *n* (%)							
Non-traumatic	52 (39)	38 (29)	14 (11)	0.408	19 (14)	33 (25)	0.410
Traumatic	80 (61)	53 (40)	27 (26)		35 (27)	45 (34)	
Level of SCI, *n* (%)							
Cervical	48 (37)	32 (24)	16 (12)	0.810^a^	18 (14)	30 (23)	0.693^a^
Thoracic	70 (54)	50 (38)	20 (15)		31 (24)	39 (30)	
Lumbar	12 (9)	9 (7)	3 (2)		4 (3)	8 (6)	
Missing	2 (2)	0 (0)	2 (1)		1 (1)	1 (1)	
AIS, *n* (%)							
A	55 (42)	41 (31)	14 (11)	0.233^a^	24 (18)	31 (23)	0.585^a^
B	11 (8)	5 (4)	6 (5)		5 (4)	6 (5)	
C	19 (15)	12 (9)	7 (5)		5 (4)	14 (11)	
D	45 (35)	33 (25)	12 (9)		19 (14)	26 (20)	
Missing	2 (2)	0 (0)	2 (1)		1 (1)	1 (1)	
Mobility status, *n* (%)							
Non ambulatory	101 (77)	69 (52)	32 (24)	0.780	41 (31)	60 (45)	0.894
Ambulatory	31 (23)	22 (17)	9 (7)		13 (10)	18 (14)	

Chi -Squared test used for categorical variables. Percentages are calculated by the total number of fallers.

*P* values ≤ 0.05 were considered significant.

*IQR* interquartile range, *AIS* association impairment scale.

^a^Fisher test exact used.

^b^Binary logistic regression.

Forty-one (31%) participants experienced more than one fall (i.e., referred to as recurrent fallers). Most recurrent fallers fell twice (*n* = 23); this was followed by three falls (*n* = 11); four falls (*n* = 4); five falls (*n* = 2); six falls (*n* = 1); and seven falls (*n* = 1). Seventy-eight (59%) participants had at least one fall with a consequence (i.e., consequential fall) including subjective (pain and psychological) and objective (laceration, soft tissue injury, fracture, and head injury) consequences and 11 (8%) participants experienced two or more consequential falls. Table 1 shows characteristics of people who fell once (single fallers) compared to recurrent fallers and people who had no consequences from their fall compared to those with consequences. Univariate analyses found no significant differences in characteristics of single versus recurrent fallers, therefore a multivariate regression model was not generated to predict recurrent fallers. Univariate analyses found a significant relationship between consequential fallers and older age (B = 0.024, S.E = 0 .011, Wald = 5.018, df = 1, *p* = 0.025) and female gender (χ^2^ (1)* = 4.961, p* = 0.026). In the multiple logistic regression model (Table 2), females were approximately 3.5 times more likely to have a consequential fall (Wald χ ^2^ (1) = 5.809, *p* = 0.016, OR = 3.581,95% CI, 1.269 to 10.103) and the odds of having a consequential fall increased by approximately 3.8% for each increased year in age (Wald χ ^2^ (1) = 8.371, *p* = 0.004, OR = 1.038,95% CI, 1.012 to 1.064*)*.

**Table 2:** Regression analysis of falls that were consequential vs non-consequential.

Participant characteristics	Multivariate logistic regression analysis – Generalised linear model (weighted)			
	B	OR	CI	*p*
Age	0.037	1.038	1.012–1.064	0.004
Gender	1.276	3.581	1.269–10.103	0.016

Fall characteristics	Multivariate logistic regression analysis - Generalised linear model (weighted)			
	B	OR	CI	*p*
Day of the week – Sunday (yes/no)	−1.630	0.196	0.061-–0.630	0.006
Witnessed by staff (yes/no)	−0.377	0.686	0.284–1.659	0.403
Activity – transfers (yes/no)	1.411	4.100	1.706–9.856	0.002
Location – bedspace (yes/no)	0.269	1.309	0.412–4.153	0.648

### Characteristics of falls

There were a total of 207 falls during the 5-year period. Table 3 reports the circumstances of falls. Falls occurred throughout participants’ admissions, from as early as two days after admission to as late as one day before discharge. Some falls occurred while accessing the community (for leisure or in preparation for discharge home) with the earliest fall in the community recorded 16 days after admission. Falls occurred mostly during the day (68%) and most were unwitnessed (72%). Sunday was the most common day in which falls occurred.

**Table 3:** Description of falls and circumstances of consequential vs non-consequential falls.

		Consequence		
Fall variable, *n* (%)	All Falls	Non-consequential	Consequential	*p*
	207	109 (53)	98 (47)	
Day of the week, *n* (%)				
Monday	31 (15)	15 (7)	16 (8)	0.033
Tuesday	20 (10)	13 (6)	7 (3)	
Wednesday	33 (16)	17 (8)	16 (8)	
Thursday	29 (14)	17 (8)	12 (6)	
Friday	32 (15)	20 (10)	12 (6)	
Saturday	26 (13)	17 (8)	9 (4)	
Sunday	36 (17)	10 (5)	26 (13)	
Month of the year, *n* (%)				
Jan	16 (8)	7 (3)	9 (4)	0.445^a^
Feb	17 (8)	11 (5)	6 (3)	
Mar	24 (12)	17 (8)	7 (3)	
Apr	22 (11)	15 (7)	7 (3)	
May	20 (10)	8 (4)	12 (6)	
Jun	18 (9)	9 (4)	9 (4)	
Jul	13 (6)	6 (3)	7 (3)	
Aug	14 (7)	6 (3)	8 (4)	
Sep	12 (6)	5 (2)	7 (3)	
Oct	8 (4)	4 (2)	4 (2)	
Nov	18 (9)	7 (3)	11 (5)	
Dec	25 (12)	14 (7)	11 (5)	
Year, *n* (%)				
2016	36 (17)	21 (10)	15 (7)	0.489
2017	36 (17)	16 (8)	20 (10)	
2018	49 (24)	26 (13)	23 (11)	
2019	49 (24)	23 (11)	26 (13)	
2020	37 (18)	23 (11)	14 (7)	
Season, *n* (%)				
Summer	58 (28)	32 (15)	26 (13)	0.244
Autumn	66 (32)	40 (19)	26 (13)	
Winter	45 (22)	21 (10)	24 (12)	
Spring	38 (18)	16 (8)	22 (11)	
Time of Day – nursing shift, *n* (%)				
Morning 7-3 pm	97 (47)	52 (25)	45 (22)	0.941
Afternoon 3–11 pm	80 (39)	42 (20)	38 (18)	
Night 11–7 am	30 (14)	15 (7)	15 (7)	
Time of day - 6 hourly blocks, *n* (%)				
Early morning 12–6 am	18 (9)	8 (4)	10 (5)	0.604
Morning 6–12 pm	68 (33)	34 (16)	34 (16)	
Afternoon 12–6 pm	73 (35)	38 (18)	35 (17)	
Evening 6 pm – 12 am	48 (23)	29 (14)	19 (9)	
Time in admission when fall occurred, *n* (%)				
1st third	56 (28)	34 (16)	22 (11)	0.091
2nd third	78 (38)	34 (16)	44 (21)	
3rd third	69 (33)	40 (19)	29 (14)	
Missing	4 (2)	1 (1)	3 (1)	
Witnessed by staff, *n* (%)				
Yes	51 (25)	34 (16)	17 (8)	0.014
No	150 (72)	70 (34)	80 (39)	
Missing	6 (3)	5 (2)	1(1)	
Activity completed at time of fall, *n* (%)				
Transfer	61 (29)	44 (21)	17 (8)	0.010^a^
Sitting	61(29)	24 (12)	37 (18)	
Wheeling	46 (22)	21 (10)	25 (12)	
Bed	12 (6)	7 (3)	5 (2)	
Sit to stand	11 (5)	5 (2)	6 (3)	
Walking	8 (4)	3 (1)	5 (2)	
Standing	4 (2)	2 (1)	2 (1)	
Missing	4 (2)	3(1)	1(1)	
Location, *n* (%)				
Bedspace	89 (43)	58 (31)	31 (15)	0.008
Bathroom	36 (17)	14 (7)	22 (11)	
Community	28 (14)	11 (5)	17 (8)	
Hospital grounds	26 (13)	9 (4)	17 (8)	
Common areas (lounge, dining, hallway, therapy)	20 (10)	11 (5)	9 (4)	
Missing	8 (4)	6 (3)	2 (1)	
Locations, *n* (%)				
Princess Alexandra hospital grounds	171 (83)	92 (44)	79 (38)	0.154
Community	28 (14)	11 (5)	17 (8)	
Missing	8 (4)	6 (3)	2 (1)	

Chi-Squared test used for categorical variables. *p* values ≤ 0.05 were considered significant.

^a^denotes Fisher test exact used.

Percentages are calculated by the total number of falls.

Transfer – transferring from one surface to another. e.g. Bed to shower commode transfer.

Sitting – includes sitting on shower commode, wheelchair and end of bed e.g. trying to pick up something from the ground while in sitting.

Wheeling – mobilising in a wheelchair e.g. going over a speedbump.

Bed – from bed, not including when transferring e.g. fall from bed when delirious.

Sit to stand – standing from a surface not including when transferring e.g. when standing up to put away clothes.

Walking – ambulating plus or minus a walking aid e.g. walking to the bathroom.

Standing – not mobilising or transferring e.g. fall when standing to complete task in therapy.

**Fig. 1:**
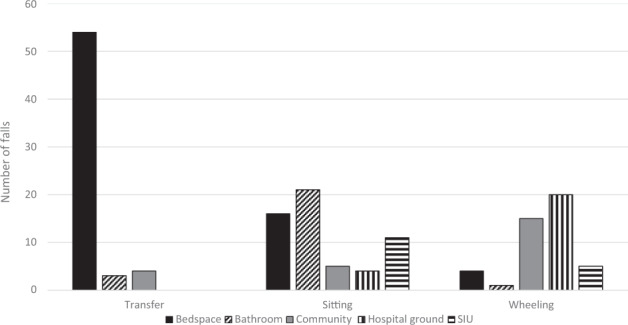
The three most common activities completed at the time of the fall and locations. SIU Spinal injuries unit.

Figure 1 shows the activities at the time of the falls and the locations where falls occurred. The activities most engaged in at the time of falling were transferring and sitting. The most common location of falls was the bedspace with falls occurring during transfers between wheelchair and bed. Falls while sitting occurred doing activities such as picking items up from the floor, completing bowel therapy, reaching for items (e.g. phone charger), putting clothes away in cupboards and reaching down to wash self. Falls while wheeling occurred mostly off the ward in the community or on hospital grounds and were most commonly attributed to environmental factors such as inclines and gutters.

Of the 207 total falls, 116 (56%) were experienced by the 41 recurrent fallers. Of the recurrent fallers, 22 (54%) fell more than once in the same location and 26 (63%) fell more than once doing the same activity. Twelve of the recurrent fallers (29%) fell more than once in the same location, while completing the same activity. Recurrent falls occurred mostly in sitting (*n* = 37 falls, 32%), transferring (*n* = 33, 28%) and wheeling (*n* = 26, 22%). Locations of recurrent falls were mostly in the bedspace (*n* = 54 falls, 47%), bathroom (*n* = 20, 17%), community (*n* = 15, 13 %) and hospital grounds (*n* = 14, 12%).

**Fig. 2:**
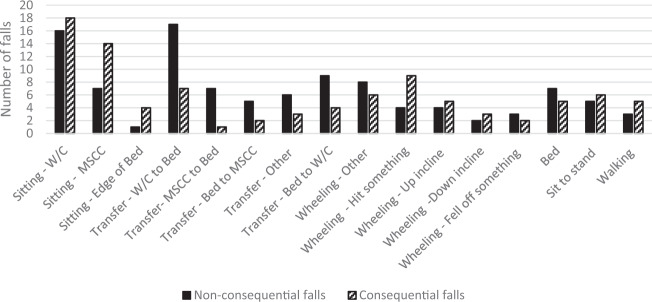
Consequential falls compared to non-consequential falls for each activity. W/C wheelchair, MSCC mobile shower commode chair.

Nearly half of the total falls, 98 (47%) were consequential. The majority (*n* = 93, 95%) had minor consequences including pain (*n* = 45, 44%), lacerations (*n* = 43, 44%), soft tissue injuries (*n* = 22, 22%), psychological consequences (*n* = 6, 6%), and other which included headache, change to taste, difficulty sleeping, haematuria and drowsiness (*n* = 6, 6%). Serious consequences occurred from five falls (2%) in five different locations and included four fractures (lower limb and nose) and one head injury. These falls occurred mainly with male participants (*n* = 4, 80%) who only fell once (*n* = 4, 80%). Their falls were mostly unwitnessed by staff (*n* = 4, 80%), occurred mostly from sitting (*n* = 4, 80%). Figure 2 shows the comparison of consequential falls versus non–consequential falls for each activity.

Table 3 shows the comparison between characteristics of non-consequential and consequential falls. Consequential falls had a significantly greater proportion of falls unwitnessed by staff (χ^2^ = 6.097, df = 1, *p* = 0.014) and falls occurring on a Sunday (χ^2^ = 10.820, df = 1, *p* = 0.001). Fewer consequential falls occurred in the bedspace (χ^2^ = 11.595, df = 1, *p* = 0.001) and when completing a transfer (χ^2^ = 13.861, df = 1, *p* = 0.000). Table 4 shows the post hoc analysis of non-binary variables for non-consequential versus consequential falls. Other variables that appeared different between non-consequential and consequential falls but were not statistically significant included falls occurring in March and April, in 2020, in autumn, and in the first third of admission.

**Table 4:** Post hoc analysis of non-binary variables for consequential vs non-consequential falls.

		Consequence		
Fall variable *n* (%)	All falls	Non-consequential	Consequential	*p*
	207	109 (53)	98 (47)	
Day of the week, *n* (%)				
Monday	31 (15)	15 (7)	16 (8)	0.606
Tuesday	20 (10)	13 (6)	7 (3)	0.245
Wednesday	33 (16)	17 (8)	16 (8)	0.886
Thursday	29 (14)	17 (8)	12 (6)	0.488
Friday	32 (15)	20 (10)	12 (6)	0.225
Saturday	26 (13)	17 (8)	9 (4)	0.165
Sunday	36 (17)	10 (5)	26 (13)	0.001
Activity completed at time of fall, *n* (%)				
Transfer	61 (29)	44 (21)	17 (8)	0.000
Sitting	61(29)	24 (12)	37 (18)	0.016
Wheeling	46 (22)	21 (10)	25 (12)	0.311
Bed	12 (6)	7 (3)	5 (2)	0.662
Sit to stand	11 (5)	5 (2)	6 (3)	0.644
Walking	8 (4)	3 (1)	5 (2)	0.483^a^
Standing	4 (2)	2 (1)	2 (1)	1.000^a^
Missing	4 (2)	3(1)	1(1)	
Location, *n* (%)				
Bedspace	89 (43)	58 (31)	31 (15)	0.001
Bathroom	36 (17)	14 (7)	22 (11)	0.088
Community	28 (14)	11 (5)	17 (8)	0.154
Hospital grounds	26 (13)	9 (4)	17 (8)	0.061
Common areas (lounge, dining, hallway, therapy)	20 (10)	11 (5)	9 (4)	0.760
Missing	8 (4)	6 (3)	2 (1)	

Chi -Squared test used for categorical variables.

^a^denotes Fisher test exact used.

*p* values ≤ 0.01 were considered significant.

Percentages are calculated by the total number of falls.

In the multiple logistic regression model (See Table 2), falls on a Sunday were approximately 5 times more consequential compared to falls on any other day of the week (Wald χ^2^ = 7.472, df = 1, *p* = 0.006, OR = 0.196, 95% CI, 0.061 to 0.630). Falls while transferring were approximately 4 times less likely to be consequential compared to falls while completing other activities (Wald χ^2^ (1) = 9.945, *p* = 0.002, OR = 4.100, 95% CI, 1.706 to 9.856). Non-significant variables have also been reported in Table 2 for interest.

## Discussion

The purpose of this paper was to describe persons who fall in inpatient SCI rehabilitation and the falls they experience to inform future fall prevention approaches. The first aim was to describe characteristics of participants who fall during inpatient SCI rehabilitation. Over the 5-year study period, 23% of inpatients had a documented fall during their admission with 41% experiencing recurrent falls. Of those who fell, 59% experienced a negative consequence such as pain, an injury, or psychological impact. These rates of consequential falls and serious injury are similar to those found in recent studies on persons with SCI living in the community [8–11, 13]. This finding shows that, even while in the supervised and supported hospital inpatient setting, individuals with SCI are at risk of falls with negative consequences and highlights the need for fall prevention during inpatient rehabilitation.

The second aim was to investigate factors associated with being a recurrent faller. Of the 41 recurrent fallers 29% experienced the same fall in the same location or while doing the same activity, in some cases up to four times. No variables were identified as significantly associated with being a recurrent faller in our study; however, in research with a SCI sample in the community, Jorgensen et al. [13] found being ambulant, being able to get up off the ground, and exercising 30 min once a week were significantly associated with being a recurrent faller, with the odds decreasing with age. In addition to participants being community-dwelling, their study included a larger proportion of ambulatory participants which may account for the difference in results. The lack of significant factors associated with recurrent falls in our study suggests all fallers may be considered potential candidates for further falls, and therefore managed accordingly. Preventing recurrent falls is potentially a meaningful target for fall prevention in which experiences of falling could be used to facilitate awareness and learning about fall prevention and put preventative measures in place. Post-fall huddles were explored by Jones et al. [21] and found to have a moderate effect on reducing repeat falls. Their effectiveness and that of other approaches to minimise recurrent falls required further prospective investigation.

In relation to the third aim, which was to identify characteristics of participants who experienced consequential falls, older age and female gender were significant, suggesting older females are at greatest risk of experiencing harm because of a fall. One possible explanation for this is that women generally report pain more frequently than men and have greater pain sensitivity [22] and pain was considered a consequence in this study. Further to this, it may be more socially acceptable for females to report pain [22]. Considering this, the significant finding for gender may be related to a reporting bias and not reflect actual consequence.

In addressing the fourth aim of describing circumstances and consequences of falls on the inpatient SIU we found falls occurred at all stages throughout the admission period. Transfers were the most common activity that resulted in a fall and the most common location was the bedspace, which is a similar finding to previous research on neurorehabilitation wards [23, 24]. More falls occurred when transferring to and from the wheelchair compared to a shower commode, which may be due to the frequency of transfers to and from wheelchairs, or it could be that patients are more supported with showering and therefore supported with transfers reducing the number of falls. Consequences of falls included pain, psychological consequences, lacerations, soft tissue injury, fractures, and head injury, and were reported in just under half (47%) of all falls although only a small proportion (5%) were serious injuries. Given most falls only resulted in minor consequences, it would be interesting to hear patients’ perceptions about the personal significance of these falls, and whether they represent an acceptable risk as part of regaining independence in daily activities. Future studies which explore whether there are long-term impacts of consequential falls, such as falls that cause pain would be of benefit to clinicians aiming to affirm fall prevention practices.

The final aim was to identify variables associated with consequential falls. Transfers were significantly less likely to be associated with consequence compared to all other activities. Compared with other activities, such as wheeling, transfers are usually completed indoors, with a plan, require a short period of concentration, and are often witnessed by staff (40% in the current study). These factors may have contributed to why falls during transfer resulted in significantly less consequences than falls during other activities. This is consistent with the finding of Zhao et al. [25] who reported if falls were witnessed, they resulted in less harm, compared to unwitnessed falls.

Falls on Sundays were more likely to have consequences compared to other days of the week. Most of the falls on Sundays (30/36) were unwitnessed by staff which may be due to almost half (*n* = 14) of the falls occurring off the ward. The presence of staff to assist and supervise patients while on the ward and when first accessing the community may allow patients to practice skills more safely and increase independence without experiencing consequences. Unlike significant participant characteristics (age and gender) which are non-modifiable, persons with SCI can be informed of additional risks of performing tasks unwitnessed which may influence the number of consequential falls experienced.

The retrospective nature of this study is a limitation because it relies on health professionals’ reports and documentation of falls at the time to be detailed and correct. While it is known falls can be underreported, especially those falls that did not result in injury [26], previous research has confirmed using incidence reports is an appropriate way to gather data about the circumstances of patients’ falls [27]. The use of the medical record meant there was only a small amount of data missing related to the recorded falls. Another limitation is that the study did not have a comparison group that did not fall during their admission, therefore not allowing for identification of predictor variables. The data analysis in the multivariate falls model included derived variables established during post hoc analysis of univariate variables although these variables required a harsher significant *p*-value of 0.01. Also, while most models assume the events are independent, this study included participants who had multiple falls. These included falls violated the assumption of falls being independent. Rather than exclude the data (75 falls, 36%) we created a variable which was single vs recurrent and found there was no significance for recurrence and severity and analysed the falls on the assumption that falls are independent. Even with this additional test we cannot exclude there may be a recurrence bias in the analysis of falls.

Future research in the inpatient rehabilitation setting comparing patients who fall during admission and those who do not would provide additional information to guide targeted fall prevention with this population. Although there has been previous research on consumer perspectives of falls with persons living in the community [28, 29] to our knowledge the patients’ perspective of falls and fall prevention has not been explored in the inpatient setting. Prospective research exploring this perspective as well as, the long-term impact of consequences of falls (i.e., increased length of stay, pressure areas) may be beneficial to determine the true magnitude of this issue.

Based on the findings from this study we recommend the focus for fall prevention in SCI rehabilitation considers the following:

1. Fall prevention needs to occur throughout the inpatient admission.
2. Fall prevention needs to focus on preventing falls on the ward while also providing inpatients with education on the risk of falls and methods to prevent falls prior to accessing the community during their admission. Consideration needs to be given to the level of support required by staff, particularly in initial community access.
3. Patients should be informed of the additional risks of sustaining injuries from falls when performing activities unwitnessed.
4. Inpatient units should be aware that Sundays are associated with increased risk of falls with physical and psychological harm and consider implications for staffing and supervision of patients.

In conclusion, this study contributes to the growing body of research on falls within the SCI population. SCI is a lifelong disability with management of falls required early in inpatient admissions [5, 30]. This study found that approximately one third of inpatients with SCI experience a fall and a quarter of these fell more than once. They experience subjective and objective consequences to these falls such as pain, psychological consequences, lacerations, soft tissue injury, fractures, and head injury. To understand predictors of these falls and the true impact of falls for consumers further research is recommended, as are intervention studies to explore the delivery and effectiveness of fall prevention programs in inpatient SCI rehabilitation.

## Data Availability

The datasets generated and analysed during the current study can be provided on reasonable request.
